# What will membrane vesicles (MVs) bring to bacterial communication?

**DOI:** 10.1264/jsme2.ME3203rh

**Published:** 2017-09-27

**Authors:** Masanori Toyofuku, Nobuhiko Nomura

**Affiliations:** 1 Department of Life and Environmental Sciences, University of Tsukuba Tsukuba, Ibaraki 305–8572 Japan

Bacteria can interact through extracellular membrane vesicles (MVs) that have several functions, including protection against antimicrobial agents, toxin delivery to host cells and horizontal gene transfer (28). In *Pseudomonas aeruginosa*, MVs carry a quorum-sensing molecule that alter the recipients’ gene expression (13). *P. aeruginosa* produces two types of *N*-acyl-L-homoserine lactone (AHL), which are *N*-butyryl-L-homoserine lactone (C4-HSL) and *N*-(3-oxododecanoyl)-L-homoserine lactone (3-oxo-C12-HSL), and a multifunctional signal, 2-heptyl-3-hydroxy-4-quinolone (PQS) that is structurally distinct to the AHLs (10, 24). PQS but not the AHLs are associated with MVs, mainly due to the hydrophobicity of the signal molecules (13, 23). A recent study showed that a long chain AHL, *N*-hexadecanoyl-L-homoserine lactone (C16-HSL) is associated with MVs produced in *Paracoccus denitrificans* and enables the signal to solubilize into the aqueous environment (26).

AHLs are the most common quorum-sensing molecule to date, and are produced in more than 200 Gram-negative bacterial species (14, 32). The *N*-acyl side chain of AHLs typically contains 4 to 18 carbons that largley affect the hydrophobicity of the molecule. It has been indicated that most of the hydrophobic long-chain AHLs partition with the cell envelope (3, 12, 19, 32). Given the low solibility in water, it was uncertain how these long chain AHLs are relesed and solubilized in aquetous environements and thus serve as a cell-to-cell signal. The MV study in long-chain AHLs and *P. denitrificans* reveals that the MV delivery of quorum sensing signal is not limited to a specific case of the PQS and *P. aeruginosa* while could be more general phenomena. By quantifying the threshold concentration of C16-HSL that can trigger the quorum sensing response in *P. denitrificans*, and the amount of C16-HSL associated with one MV, only one MV seems to be quantitatively sufficient enough to induce related gene expression in a cell (26). This finding provides a new model in quorum sensing. The classic quorum sensing model is based on an analog accumulation and homogenous distribution of the signal in the environment. Once the signal reaches a critical concentration, it synchronously induces the quorum sensing response of the majority of the cells (6). In contrast, the MV-driven quorum sensing would be a binary signaling mechanism (26). The signals could be heterogeneously distributed among cells through MVs and the quorum sensing response would be induced in only the cells that contact with MVs. Furthermore, the packaging of signals in MVs would be an effective and stable signal transfer in natural environments to avoid the dilution below the threshold concentration.

Another feature of cell-to-cell signaling by MVs is the traffic control of the signals. Free signals will be conveyed randomly to the surrounding bacterial cells while MV-bearing signals would be selectively delivered to the cells that have certain affinity to MVs and therefore, the traffic control of signals becomes possible. Indeed, MVs produced by *P. denitrificans* were delivered better to *P. denitrificans* than other bacterial species tested (26). For example, using a quorum sensing reporter strain, it was shown that *P. denitrificans* responds equally to free C16-HSL and MV-associated C16-HSL, whereas *P. aeruginosa* could respond to free C16-HSL but not sufficiently to MV-associated C16-HSL (26). The specific delivery of signals would be important in natural microbial communities usually composed of heterogeneous microbial populations. Similar results were observed in another bacterial species of *Buttiauxella agrestis*, of which MVs were specifically delivered to the same species due to the physicochemical properties of surface interaction (22). The surface of *B. agrestis* cell posses significantly lower zeta potential compared to those of other bacteria. The low zeta potential and the hydrodynamic diameter of *B. agrestis* cells lead to low energy barrier between *B. agrestis* and its MVs, which can be calculated based on the Derjaguin–Landau–Verwey–Overbeek (DLVO) theory (8). The study indicates that DLVO theory is applicable to estimate the interaction of the cells and MVs. Further detailed study, however, showed that DLVO theory fail to explain some of the MV-bacterial interactions observed (22), indicating that other mechanisms would control the MV-bacterial interactions. There are many surface proteins known to be involved in autoaggregation of the cells, which may be also involved in the interactions. By changing the composition of surface proteins of MVs, MVs can be trafficked to certain types of cells (7). For these purposes, molecular tools to decorate the surface with different proteins have been developed (2, 5).

To use MVs as a platform for application, it will be vital to establish methods for mass production of MVs. There has been progress in understanding how MVs are formed in bacteria. In Gram-negative bacteria, it is proposed that MVs are formed by blebbing of the outer membrane and several mechanisms are hypothesized to induce the blebbing of the membrane (20). Recently, a new model has been proposed based on the observation that MVs can be formed through explosive cell lysis in *P. aeruginosa* (30). Live cell imaging of explosive cell lysis tracked that the shattered membranes derived from cell lysis immediately round up and form MVs. It was also found that the MVs formed through explosive cell lysis randomly encapsulate DNA (30), which may explain why MVs often carry cytoplasmic components as their cargo. The direct triggering of explosive cell lysis is a peptidoglycan-degrading enzyme, endolysin, which is encoded in a cryptic prophage region in the *P. aeruginosa* genome (15). Under normal conditions, the gene expression is suppressed but is enhanced in the cells under stressed conditions including anoxoic conditions and biofilm formation (29, 30). Under these conditions, the majority of MVs were derived from the explosive cell lysis. In addition, the explosive cell lysis provides extracellular DNA, one of the main extracellular matrix components in the biofilms (30). Simultaneous release of MVs and the matrix will explain why MVs are so abundant in the biofilm matrix (27). Since endolysin-induced explosive cell lysis occurs in the sub-populaiton of the cells, the remaining cells can use the released materials as public goods. The finding further suggests that endolysin is a universal trigger for MV formation.

Endolysin is a well-conserved enzyme that is typically involved in the release of dsDNA phages (16). More recently, it has been shown that endolysin induces MVs in Gram-positive bacteria through a distinct mechanism to explosive cell lysis (25). As compared to Gram-negative bacteria, Gram-positive bacteria possess the thick cell wall surrounding the cytoplasmic membrane and it remained a mystery how MVs are released through this thick cell wall (4). A study using *Bacillus subtilis*, revealed that endolysin opened holes in the cell wall, through which MVs are formed presumably due to turgor pressure, and are eventually released (25). The cells that released MVs eventually die, but similar to the observation in *P. aeruginosa*, endolysin-induced MV formation occurs in the sub-population of the cells. Endolysin that is simultaneously released with MVs can also induce MVs of nearby cells by degrading their cell walls from outside. This is consistent with the results observed in previous studies using endolsyin as antibacterial agents. The endolysin-treated cells often show membrane protrusions (11). These observations indicate that endolysin can induce formation of MVs in a wide range of Gram-positive bacteria. Given the high abundance of phages in natural environments (9, 17, 18, 21), the cell death may be a major source for MVs.

The findings that cell death is involved in MV formation renew our concept of cell death in microbial communities (1). MVs were considered to be produced only from growing cells but not from lysing cells. Key experiments are live imaging of microbial communities, particularly biofilm-forming microbial communities, with high-resolution visualization and diagnosis of live and dead at single cell level. MVs derived from cell lysates possess biological functions, suggesting that they are not mere cell debris. In *B. subtilis*, cell lysates containing MVs are able to mediate exchange of a phage receptor to phage resistant cells (31). This enables the phage to invade the resistant cells and would have impact on the horizontal gene transfer among bacteria. The MV formation in *P. denitrificans* is also induced by DNA-damaging stress, which resulted in the increased release of C16-HSL in the extracellular milieu (26). The involvement of RecA in this process suggests that MVs are formed through explosive cell lysis (26, 30), and implies that prophages can stimulate cell-to-cell communication through MV formation, which is currently under investigation. We have known that bacteria can communicate with each other for years (6). Now, we are going to understand that bacteria communicate in a digital language, send “messages in a bottle” and even leave dying messages for their follows, enemies and descendants.
